# Host Preference between Symbiotic and Aposymbiotic *Aphis fabae*, by the Aphid Parasitoid, *Lysiphlebus ambiguus*

**DOI:** 10.1673/031.011.8101

**Published:** 2011-07-02

**Authors:** Rui-Xia Cheng, Ling Meng, Nickolas J Mills, Baoping Li

**Affiliations:** ^1^ Department of Entomology, Nanjing Agriculture University, Nanjing 210095, China; ^2^ Office of Landscape Management, Kunshan city, Jiangsu Province, China; ^3^ Department of Environmental Science, Policy and Management, University of California, Berkeley, CA 94720-3114, USA

**Keywords:** Aphidiidae, bacterial symbiosis, *Buchnera aphidicola*, host choice, host instar, host suitability, sex ratio

## Abstract

Few empirical studies have directly explored the association between *Buchnera aphidicola* (Enterobacteriales: Enterobacteriaceae), the primary endosymbiont of aphids, and the life history strategies of aphid parasitoids. A series of paired-choice experiments were conducted to explore the preference of the parasitoid *Lysiphlebus ambiguus* Halliday (Hymenoptera: Aphididae) for symbiotic and aposymbiotic *Aphis fabae* Scopoli (Hemiptera: Aphididae) and the suitability of these hosts for parasitoid development. When given a choice between symbiotic and aposymbiotic aphids of the same instar, the parasitoid significantly preferred symbiotic over aposymbiotic aphids only during the later instars (L_4_ and adult). The suitability of aposymbiotic aphids for parasitoid development was equal to that of symbiotic aphids in terms of survivorship and sex ratio, but was significantly lower than that of symbiotic aphids for L_4_ and adult instars in development rate and/or female adult size. When given a choice between similar-sized symbiotic L_2_ and aposymbiotic L_4_ aphids, the parasitoid preferred the former. No significant differences in preference or host suitability were demonstrated when the parasitoid was given a choice between different instars of aposymbiotic aphids. While parasitoid lifetime fecundity increased with aphid instar at the time of oviposition, there was no significant influence of previous development from symbiotic versus aposymbiotic aphids. These results suggest that while *L. ambiguus* can discriminate between symbiotic and aposymbiotic *A. fabae* during later instars and when the aphids are of a similar size, the primary endosymbiont is not needed for successful parasitoid development; and its absence only compromises parasitoid growth reared from later instar aposymbiotic host.

## Introduction

Symbiosis in aphids (Sternorrhyncha: Aphidoidea) has been extensively studied (Douglas 1998; Ferrari et al. 2004; Moran et al. 2005; Chandler et al. 2008). The majority of obligate aphid symbionts are bacteria, the primary obligate y-proteobacterium *Buchnera aphidicola* (Enterobacteriales: Enterobacteriaceae), located in specialized cells called mycetocytes or bacteriocytes that are loosely aggregated through the abdominal haemolymph (see Douglas 1998 for a full review). Many aphids also have a variety of secondary bacterial symbionts that are either associated with the bacteriocytes of *Buchnera* or found in the haemolymph (Moran et al. 2005). The impact of the loss of the primary symbiont has also been well—documented in aphids (Wilkinson 1998). For example, bacteria-free or aposymbiotic pea aphids *Acyrthosiphon pisum*, produced by mild antibiotic therapy, have a reduced growth rate, attain a lower adult size, and are reproductively sterile (Sasaki et al. 1991; Douglas 1992). The principal conclusion from research on nutritional interactions in the aphid*-Buchnera* symbiosis is that the bacteria provide aphids with essential amino acids (Douglas 1998; Gunduz and Douglas 2009). However, not only does the number of primary bacteriocytes vary with temperature (Ohtaka and Ishikawa 1991; Li and Li 2006; Chen et al. 2009) and aphid development (Lamb and Hinde 1967; Douglas and Dixon 1987; Li and Li 2006; Bermingham et al. 2009), but also the function of the primary symbionts can be influenced by host plant (Wilkinson et al. 2001) and heat shock (Russell and Moran 2006; Dunbar et al. 2007).

In addition, a link between parasitoid development and primary symbionts in aphids has been supported by several lines of evidence. Pennachio et al. (1999) found that the parasitoid *Aphidius ervi* performed poorly in pea aphids experimentally deprived of their symbiotic bacteria; Rahbé et al. (2002) demonstrated that essential amino acid synthesis by *Buchnera* is selectively preserved or promoted in parasitized aphids; and Cloutier and Douglas (2003) demonstrated that the number and biomass of bacteriocytes were elevated in parasitized pea aphids. It has also been shown that secondary symbionts of aphids can be strongly associated with resistance to aphidiine parasitoids (Ferrari et al. 2004; Oliver et al. 2005). For pea aphids, a toxin-encoding bacteriophage that is associated with the secondary symbiont *Hamiltonella defensa* appears to be the mechanism for resistance to *A. ervi* (Oliver et al. 2009).

In a previous study a decline was observed in female proportions at emergence of offspring adults of *Lysiphlebus ambiguus* Halliday (Hymenoptera: Aphididae) in relation to host instar at the time of oviposition for cohorts of *Aphis fabae* Scopoli (Hemiptera: Aphididae) reared at high temperatures (Xu et al. 2008). It was suggested that differential mortality might have caused the decline in sex ratio as a result of disruption of the primary symbiont at high temperature. This hypothesis was derived from the earlier observation that aposymbiotic pea aphids were found to be less suitable hosts for the growth and development rate of juvenile parasitoids (Pennachio et al. 1999). However, studies of *A. ervi* by Falabella et al. (2000) and Cloutier and Douglas (2003) showed that host regulation of parasitoid larvae can ameliorate such stress to some extent. The study of *L. ambiguus* by Chen et al. (2010) even suggests that parasitoid larvae might be able to compensate for the detrimental effect associated with disruption of primary endosymbiotc bacteria in aposymbiotic aphids. These studies suggest that host quality in aphids for parasitoid offspring development can be closely associated to endosymbiotic activity. Thus the realization of any deleterious impacts on juvenile parasitoids is contingent upon either the ability of female parasitoids to discriminate at oviposition between host aphids with and without normally functioning primary symbionts, or the ability of parasitoid larvae to regulate host aphids with abnormally functioning endosymbionts. Until now, few empirical studies have directly explored the association between the primary endosymbiont of aphids and host preferenceperformance of aphid parasitoids.

Here, a series of preference-performance trials were conducted using an aphid—parasitoid system, *Aphis fabae/Lysiphlebus ambiguus*, to explore whether aphid parasitoids base their host choice on activity of primary endosymbiotic bacteria, and whether parasitoid larvae can compensate for the detrimental effect associated with disruption of primary endosymbiotc bacteria in aposymbiotic aphids.

## Materials and Methods

### Insect cultures

A general colony of the black bean aphid, *Aphis fabae* Scopoli (Hemiptera: Aphididae) was maintained on potted plants of the broad bean, *Vicia faba* L. (Fabales: Fabaceae), under long day conditions (20 ±2° C, 16:8 L:D) in an insectary. To obtain aposymbiotic host aphids, the primary symbiont in *A. fabae* was eliminated using the antibiotic rifampicin (MDBio Inc., www.mdbio.com.tw), administered via plants using the method of Douglas (1996) and Miao et al. (2003, 2004). The roots of 3-week-old *V. faba* plants were cleaned of excess compost by carefully washing them in tap water before transferring them to conical flasks (50 ml) with distilled water containing 200µg/ml rifampicin. Control plants were treated similarly, but transferred to flasks with antibiotic-free distilled water. One day later, these plants were infested with young apterous adult aphids, which were allowed to feed and reproduce for 5 h before being removed from the plants. The cohorts of newly-produced aphid nymphs were maintained on both the rifampicin-treated and control plants for two days. All aphid nymphs were then removed to plants in antibiotic-free distilled water.

Aphids from rifampicin-treated plants are referred to as aposymbiotic aphids, those from rifampicin-free plants as symbiotic aphids. Using the same method, the primary symbionts of the cowpea aphid *Aphis craccivora* began to degrade within 48 h at 23° C, and to breakdown within 144 h; although treated individuals were able to complete their development, development time was increased, adult size was reduced, and few were able to reproduce (Miao et al. 2004). In the current experiments, *A. fabae* on rifampicin-treated plants showed the same developmental response as *A. craccivora*, and the degradation of the primary symbiont was confirmed by dissection under light microscopy. As rifampicin appears to be selective in eliminating the primary symbiont while secondary symbionts may not be affected (Koga et al. 2007), the occurrence of the facultative bacterium *Hamiltonella defensa* was also tested. This secondary symbiont can mediate host resistance to aphidiine parasitoids (Oliver et al. 2005), and is known from populations of *A. fabae* (Moran et al. 2005). Following Douglas et al. (2006), a PCR assay was used, based on the diagnostic PABSF and 16SB1 primers for *H. defensa*, and its occurrence was not detected in the aphid colonies used in these experiments.

The aphid parasitoid *L. ambiguus* was collected in November 2005 from *A. fabae* on broad beans, *V. faba*, in Zhenjiang city (32.0° N and 118.7° E), Jiangsu Province, Eastern China. The parasitoid was reared under the same natural conditions in an insectary using *A. fabae* on potted (15 cm diameter) *V. faba* in wooden cages (45 × 45 × 50 cm) with sides of plastic 60-mesh nylon cloth. Experimental *L. ambiguus* were removed from the general culture as aphid mummies and placed in glass tubes where they could mate after eclosion. *L. ambiguus* were provided with a 50% honey solution as food, but not given access to aphids. Females used in the experiments were 24 h old, naïve, and randomly chosen from the vials (20 ± 2° C, 16:8 L:D).

### Host preference experiments

Two paired-choice trials were conducted to assess *L. ambiguus* preferences between symbiotic and aposymbiotic aphids with the control for influences of host age and body size, and another one was performed to evaluate preferences between different instars of aposymbiotic hosts. The ensuing *L. ambiguus* progeny was also observed for developmental performances. In the first choice trial symbiotic and aposymbiotic aphids of the same instar (L_2_ to adult) were simultaneously exposed to parasitism, so as to control for the confounding effect of host instar. In the second paired-choice trial that was designed to control for the effect of host body size, symbiotic L_2_ and aposymbiotic L_4_ aphids were chosen as pairs in consideration of requirements of both complete disappearance of the primary symbiont in aposymbiotic aphids and easiness of distinguishing each treatment. The confounding effect of body size was controlled for by using the analysis of covariance with body size as a covariable. In the third experiment pair-wise instars of L_1_ to L_4_ aposymbiotic aphids were subjected to parasitism.

In each of the above experiments, 20 aphids from each of the paired cohorts of aphids were introduced into a Petri-dish (5 times; 1.5 cm) on an excised bean leaf with its petiole inserted into a small glass-vial of water to keep the leaf fresh. A single female wasp was then introduced into the Petri-dish and was observed continuously for a period of 3 h to monitor probing events, after which the parasitoid was removed, and the paired cohort aphids were separated to different Petri-dishes where plant leaves were provided as food. The aphids for each treatment were distinguished based either on body size where symbiotic (larger) and aposymbiotic (smaller) of the same instar and different instars of aposymbiotic aphids were paired, or on body color where symbiotic L_2_ (pale) and aposymbiotic L_4_ (darker) aphids with similar body size were paired. The leaf was replaced when necessary and the aphids were examined daily for mummification. Mummies were collected and kept in glass tubes for emergence of adults. From previous dissections of aphids it was determined that a probing event that lasted for more than 30 seconds always resulted in egg-laying and is referred to as an effective sting. For each experiment, the number of effective stings (from direct observation), adult emergence rate (number of adults produced per effective sting), sex ratio (proportion of female progeny), developmental time (from effective sting to adult emergence in days), and adult female body size (hind tibia length in mm) were noted. Fifteen replications for each paired comparison were carried out, but were subject to slight variation owing to rare cases where no mummies were formed.

### Survivorship of *L. ambiguus* on different instars of symbiotic and aposymbiotic aphids

As the number of mummies produced in the paired-choice preference experiments was in some cases small, the sample sizes might have been inadequate for a rigorous assessment of the influence of symbiotic versus aposymbiotic aphids on the survivorship of *L. ambiguus.* Therefore an additional experiment was conducted to estimate host suitability from a comparison of the number of mummies produced from a larger number of observed effective stings. A naïve mated female parasitoid was introduced into a gelatin capsule where a single aphid was exposed to parasitism. The aphid and parasitoid were observed continuously until an effective sting of more than 30 seconds occurred, and then the aphid was removed from the gelatin capsule and reared on a potted bean plant. A total of 130–155 aphids from each instar (L_1_ through to adult) of both symbiotic and aposymbiotic cohorts were tested, from which mummy production was estimated.

### Influence of aphid symbiosis on parasitoid fecundity

In order to measure the performance of parasitoid progeny produced from both symbiotic and aposymbiotic aphids, their lifetime fecundity was estimated. Parasitoid progeny emerging from aphids attacked as L_1_– 3 were initially kept in groups in glass vials to allow mating, and then individual females were introduced into Petri-dishes with a 50% honey solution as food. The Petri-dishes were provisioned with symbiotic aphids (ca. 60) of one particular instar (with separate observations for L_1_ to adult aphids) and exposed to parasitism for 9 hours (09:00–18:00). The female parasitoids were transferred to new Petri-dishes of aphids each day. Three days after exposure to the female parasitoids the host aphids were dissected under a stereo microscope and the number of hosts parasitized was noted. The experiment was terminated when the female parasitoids had died. The lifetime fecundity of each parasitoid female was measured as the total number of host aphids parasitized. The experiment was replicated for 10 females produced from both symbiotic and aposymbiotic hosts.

### Data analysis

In consideration of the non-independence of the paired-choice experiments, the Wilcoxon paired-sample test was employed, with Bonferroni correction for multiple tests, to examine differences in number of effective stings, adult emergence rate, progeny sex ratio, development time, and adult body size of the *L. ambiguus* between symbiotic and aposymbiotic host aphid cohorts exposed to parasitism. In analyzing *L. ambiguus* preference and host suitability in the trial of paired-choices between symbiotic L_2_ and aposymbiotic L_4_ aphids, generalized linear models were fitted to the observation variables as explained by variation in symbiosis and body size of hosts. Sex ratio was defined as the proportion of females among the parasitoid progeny. After arcsine square root transformation, a two-way ANOVA was used to analyze *L. ambiguus* survivorship experiment with aphid symbiosis (symbiotic or aposymbiotic) and aphid instar (L_1_ to adult) as factors. Similarly after log transformation, a two-way ANOVA was used to analyze the lifetime fecundity of parasitoids with host rearing type (symbiotic or aposymbiotic) and host instar as factors. Data analyses were performed using R statistical software (R Development Core Team 2007).

## Results

### Host preference experiments

**Paired-choice between symbiotic and aposymbiotic aphids of the same instar.** When given a choice between two equally available host cohorts, a comparison of the number of effective stings indicates that the parasitoid showed a significant preference for symbiotic aphids over aposymbiotic aphids in the L_4_ and adult instars, but that no such preferences were found between paired cohorts for earlier instars. In the case of host suitability for parasitoid development, adult emergence rate and sex ratio did not differ significantly between symbiotic and aposymbiotic aphids for any of the instars, but development time was significantly longer for aposymbiotic aphids in the L_4_ and adult instars; and adult female size was significantly smaller for the aposymbiotic adult instar. This suggests that although sample sizes were small in some cases (Table 1), the primary endosymbiont did not affect parasitoid survivorship or sex ratio, but that parasitoid preference was influenced in those later instars where progeny development time and adult female body size were compromised in aposymbiotic aphids.

**Paired-choice between symbiotic (L_2_) and aposymbiotic (L_4_) host aphids of the same body size.** The generalized linear model with quasi-Poisson error fitted to the number of effective stings showed that under the control for host body size which was not significant, parasitoid launched significantly more attacks on symbiotic L_2_ than on aposymbiotic L_4_ aphids (effective stings: L_2_, 13.17 ± 2.33; L_4_, 8.25 ± 1.92; *p* < 0.05). There were no significant differences between symbiotic L_2_ and aposymbiotic L_4_ aphid hosts in suitability for parasitoid progeny, as measured by adult emergence rate (L_2_, 0.92 ± 0.12; L_4_, 0.93 ± 0.12) and sex ratio (female proportion: L_2_, 0.93 ± 0.13; L_4_, 0.77 ± 0.34).

**Paired choice between instars of aposymbiotic aphids.** Among all host instar-pairs of aposymbiotic aphids exposed to parasitism, the parasitoid only showed a significant preference for L_2_ over L_1_ (*p =* 0.04) and an almost significant favorite for L_1_ over L_3_ (*p* = 0.07), as measured by the number of effective stings (Table 2). There were no significant differences in host suitability for offspring development (as measured by development time, adult emergence rate, proportion of females, and female body size) between instars in any of the paired-choices examined (Table 2).

### Survivorship of *L. ambiguus* on symbiotic and aposymbiotic aphids

The mummification rate, representing the survivorship of parasitoid juveniles from oviposition to host mummification, differed significantly among host instars (Table 3, two-way ANOVA, *F*_4,50_ = 8.83, *p* < 0.001) and symbiosis types (*F*_1,50_ = 16.42, *p* < 0.001), although there was also a significant interaction (*F*_4,50_ = 3.49, *p* = 0.007). The interaction resulted from different patterns of survivorship with respect to instar for symbiotic versus aposymbiotic aphids; the pattern for the former being a general decline from L_1_ to adult with the exception of L_2_ which was lower than might be expected, while the pattern for the latter peaked at L_3_ with a decline for both earlier and later instars (Table 3). Within each instar, survivorship between symbiotic and aposymbiotic aphids did not differ for L_2_, L_3_ and adult aphids, but was significantly lower for aposymbiotic aphids for L_1_ and L_4_.

### Influence of aphid symbiosis on parasitoid fecundity

**Figure 1.**
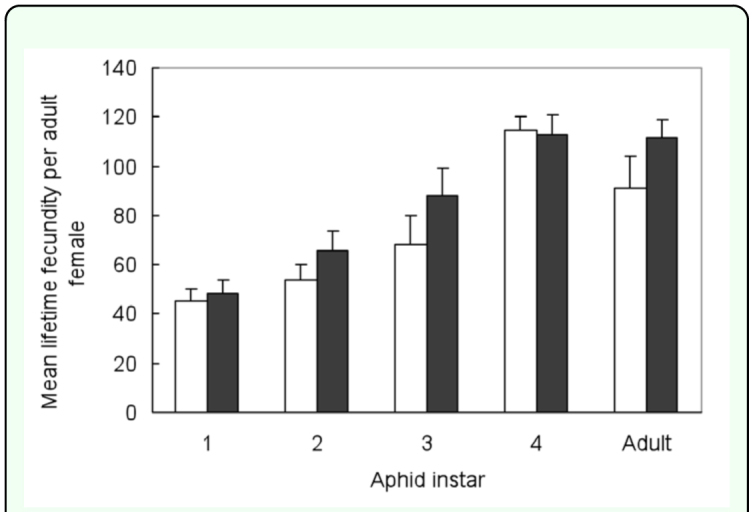
The mean lifetime fecundity (± SE) as measured by number of mummies formed of *Lysiphlebus ambiguus*, reared from either symbiotic (black bars) or aposymbiotic (white bars) *Aphis fabae*, in relation to aphid instar at the time of oviposition, where a parasitoid had access to 60 aphids for 9 h per day until end of reproduction. High quality figures are available online

The lifetime fecundity of parasitoid progeny reared from either symbiotic or aposymbiotic aphids was estimated for females in relation to host instars of symbiotic aphids for parasitization (Figure 1). The two-way ANOVA did not show a significant interaction between host instars at the time of oviposition and symbiosis of rearing hosts (two-way ANOVA, *F*_4,50_ = 0.57, *P* = 0.69), but indicated a significant influence of host instars (*F*_4,50_ = 19.07, *P* < 0.001) and symbiosis of rearing hosts (*F*_1,50_ = 4.36, *p* = 0.04). The lifetime fecundity of offspring parsitoids showed a steady increase in relation to host instars available from L_1_ to L_4_, with that for adult hosts equivalent to L_4_ hosts.

## Discussion

Numerous studies have demonstrated that the obligate bacterial endosymbiont, *Buchnera aphidicola*, provides aphids with essential amino acids (Douglas 1998; Chandler et al. 2008; Gunduz and Douglas 2009). It has also been shown for *A. pisum* that the growth and development of *A. ervi* larvae is closely linked to the activity of the primary endosymbiont (Pennachio et al. 1999; Rahbé et al. 2002; Cloutier and Douglas 2003) and to the presence of any detrimental secondary endosymbionts (Ferrari et al. 2004; Oliver et al. 2005). Thus, it is generally assumed that the reproductive success of female aphidiine parasitoids is dependent on their ability to accurately assess the suitability of a host for larval development at the time of oviposition. For an idiobiont parasitoid that stops any further host development at the time of oviposition, the decision to parasitize a host should be based on whether the quality and quantity of the nutrients available outweigh the costs in terms of time and energy (Rivero 2000). In contrast, for most koinobiont parasitoids the potential to assess host suitability is limited by the unpredictability of future host growth and survivorship following oviposition (Harvey 2005). However, although they are koinobionts, aphidiine parasitoids appear to use an intermediate development strategy in that, as for idiobiont parasitoids, both sex allocation and final adult size are strongly influenced by host size at oviposition (Mackauer et al. 1997; Li and Mills 2004; Cao and Li 2007; Xu et al. 2008).

For aphidiine parasitoids, such as *L. ambiguus*, to be able to detect, assess, and respond to host quality at oviposition they must employ cues associated with nutritional status that are not found in the hosts of other koinobiont parasitoids. Aphids differ from other phytophagous hosts in the prominent role played by symbiotic bacteria, and if these bacteria provide cues that can be used in the assessment of host quality it is expected that symbiotic aphids would be subjected to a greater number of effective stings than their aposymbiotic counterparts. Our study provides some support for this expectation. In two of the dual-choice experiments, in which either host instar or host body size were controlled, *L. ambiguus* showed a clear preference for symbiotic compared to aposymbiotic aphids, as measured by the number of effective stings. That this preference was exhibited only at the later stages of aphid development (L_4_ and adult) suggests that disruption of the primary endosymbiont through the rifampicin treatment did not cause sufficient change during the early aphid instars for the parasitoid to find a detectable difference between symbiotic and aposymbiotic aphids.

Where both preference and performance of parasitoids have been measured, there has generally been good concordance (e.g. Ode et al. 2005). In our study, when *L. ambiguus* did show a preference between symbiotic and aposymbiotic hosts in the dual choice experiments, although there were no significant differences in mummy production, adult emergence rate or progeny sex ratio (Table 1, 2) were significant reductions in either development rate and/or adult female size of the progeny (Table 1). The reduction in performance observed for *L. ambiguus* in late instar aposymbiotic *A. fabae* was much less than the 50% reduction in adult size found by Pennachio et al. (1999) for *A. ervi* developing in aposymbiotic *A. pisum* and by Miao et al. (2004) for *Lysiphlebus japonicus* developing on aposymbiotic *A. craccivora.* Nonetheless, there was some evidence from *L. ambiguus* of a linkage between preference and performance in relation to the symbiont status of their *A. fabae* hosts, and this linkage appears to be mediated through the growth rather than survivorship of the parasitoid progeny, as found by Miao et al. (2004). Such linkages do not always occur for insect parasitoids, with exceptional circumstances including threat of hyperparasitism (Ayal and Green 1993), host defense (Gerling et al. 1990), parasitoid learning (Wardle and Borden 1991), physiological constraints on egg production or oviposition (Rivero 2000), and host transfer (Henry et al. 2005).

It is perhaps surprising that parasitoid progeny survivorship was not significantly impacted by disruption of the primary endosymbiont in aposymbiotic aphids receiving the antibiotic treatment, as both the number and biomass of bacteriocytes (Cloutier and Douglas 2003) and the level of amino acid synthesis (Rahbé et al. 2002) increase in response to parasitism in normal symbiotic aphids. However, success of parasitism is not only dependent on amino acid production by the primary endosymbiont, but also on the activity of parasitoid-derived teratocytes in incorporating the amino acids into proteins (Pennachio et al. 1999; Rahbé et al. 2002). Thus there is potential for variation in the influence of aposymbiosis in aphids on success of parasitism due to the unknown quantitative contribution of the primary symbiont to aphid success (Brinza et al. 2009), and the poorly understood interaction of the primary symbiont and parasitoid teratocytes (Cloutier and Douglas 2003). It is also possible that secondary endosymbionts (other than *H. defensa*, which was not found in PCR assays) may have been present in the aposymbiotic aphids used in this study and could have played a role in compensating for the loss of the primary endosymbiont (Koga et al. 2003).

In addition, the absence of a significant difference in secondary sex ratio between symbiotic and aposymbiotic aphids (Table 1) suggests that there was neither a response in the sex ratio allocation by female *L. ambiguus*, nor any evidence for differential survivorship of the two sexes during development. Thus it seems unlikely that the decline in secondary sex ratio that was observed for symbiotic *A. fabae* reared at high temperatures in previous studies (Li and Mills 2005; Xu et al. 2008) would have been caused by heat-induced disruption of primary endosymbiont activity. While both aposymbiosis and heat stress lead to a reduction in aphid growth and heat stress reduced primary symbiont activity in *A. craccivora* (Chen et al. 2009), heat stress may also have a greater impact on host suitability for parasitoid development, as (unlike aposymbiosis) heat-stressed aphids continue to be reproductive. Thus our earlier observation of a reduction in the secondary sex ratio of *L. ambiguus* in aphids reared at high temperatures may have resulted from a reduction in host nutritional quality due to continued aphid reproduction rather than disruption of the activity of the primary endosymbiont.

The lifetime fecundity of similar-sized parasitoid progeny reared from both symbiotic and aposymbiotic aphids was equivalent irrespective of the aphid instar supplied (Figure 1). This further indicates that disruption of the primary endosymbiont through antibiotic treatment did not compromise the reproductive capacity of *L. ambiguus* reared from early instar aposymbiotic hosts. In contrast, Miao et al. (2004) found that the lifetime fecundity of *L. japonicus* was significantly reduced for females that had completed their development in aposymbiotic *A. craccivora*, although this was mediated through a reduction in adult female parasitoid size.

Thus, overall, it appears that parasitism of *A. fabae* by *L. ambiguus* is less dependent on the activity of the primary endosymbiont than is the case for *A. ervi* in *A. pisum* (Penacchio et al. 1999; Cloutier and Douglas 2003) and *L. japonicus* in *A. craccivora.* Further analysis of a broader range of aphid-parasitoid systems will be needed to clarify the extent to which the primary endosymbiont contributes to the success of parasitism in aphids.

**Table 1.**
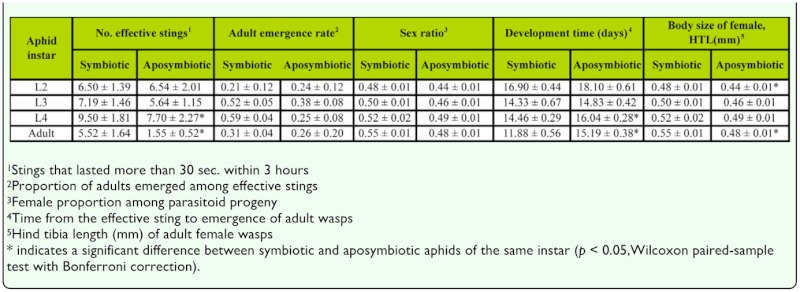
Mean (± SE) parasitoid preference (number of effective stings) of *Lysiphlebus ambiguus* and host suitability for parasitoid development when given a choice between symbiotic and aposymbiotic *Aphis fabae* of the same instar in paired-choice experiments.

**Table 2.**
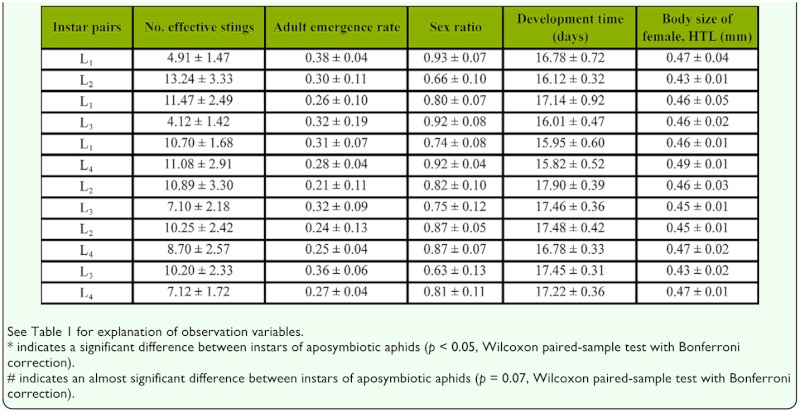
Mean (± SE) parasitoid preference (number of effective stings) of *Lysiphlebus ambiguus* and host suitability for parasitoid development when given a choice between different instars of aposymbiotic *Aphis fabae* in paired-choice experiments.

**Table 3.**
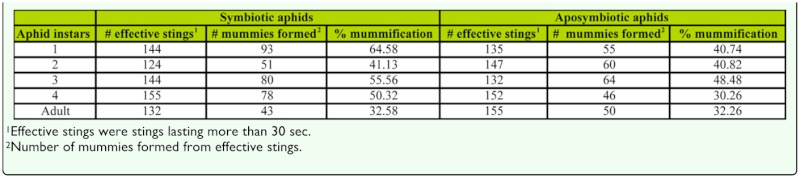
Host suitability for development of offspring parasitoids of different instars of symbiotic and aposymbiotic aphids at the time of oviposition as shown by effective stings and mummies formed.
